# A Novel bispecific T-cell engager (BiTE) targeting CD22 and CD3 has both in vitro and in vivo activity and synergizes with blinatumomab in an acute lymphoblastic leukemia (ALL) tumor model

**DOI:** 10.1007/s00262-023-03444-0

**Published:** 2023-05-29

**Authors:** Joshua F. Meckler, Daniel J. Levis, Daniel P. Vang, Joseph M. Tuscano

**Affiliations:** 1 Division of Hematology and Oncology, Department of Internal Medicine, University of California Davis School of Medicine, Sacramento, CA USA; 2 Department of Veterans Affairs, Northern California Healthcare System, Sacramento, CA USA; 3 Department of Internal Medicine, Division of Hematology and Oncology, University of California Davis Health System, 4501 X Street, Suite 3016, Sacramento, CA 95817 USA

**Keywords:** Bispecific T-cell engager, CD22, CD3, Acute lymphoblastic leukemia (ALL)

## Abstract

Immunotherapy has revolutionized cancer therapy. Two recently FDA-approved immunotherapies for B-cell malignancies target CD19, in the form of a Bispecific T-Cell Engager (BiTE) antibody construct or chimeric antigen receptor T (CAR-T) cells. Blinatumomab, an FDA-approved BiTE, binds to CD19 on B cells and to CD3 on T cells, mediating effector-target cell contact and T-cell activation that results in effective elimination of target B cells. Although CD19 is expressed by essentially all B-cell malignancies at clinical presentation, relapses with loss or reduction in CD19 surface expression are increasingly recognized as a cause of treatment failure. Therefore, there is a clear need to develop therapeutics for alternate targets. We have developed a novel BiTE consisting of humanized anti-CD22 and anti-CD3 single chain variable fragments. Target binding of the anti-CD22 and anti-CD3 moieties was confirmed by flow cytometry. CD22-BiTE promoted in vitro cell-mediated cytotoxicity in a dose and effector: target (E:T)-dependent fashion. Additionally, in an established acute lymphoblastic leukemia (ALL) xenograft mouse model, CD22-BiTE demonstrated tumor growth inhibition*,* comparable to blinatumomab. Further, the combination of blinatumomab and CD22-BiTE yielded increased efficacy in vivo when compared to the single agents. In conclusion, we report here the development of a new BiTE with cytotoxic activity against CD22^+^ cells which could represent an alternate or complementary therapeutic option for B-cell malignancies.

**Supplementary Information:**

The online version contains supplementary material available at 10.1007/s00262-023-03444-0.

## Introduction

Breakthrough immunotherapy treatments such as rituximab, and more recently CAR-T therapies, have significantly extended survival for non-Hodgkin’s lymphoma (NHL) and ALL patients. An additional treatment modality exists in bispecific antibodies (BisAbs), the concept of which dates to the 1960s when Alfred Nisonoff envisioned the potential of replacing one of the two identical antigen binding arms with a different antigen binding specificity [1]. Many BisAbs have been developed with broad therapeutic potential due to their ability to target a variety of surface antigens that distinguish different cell or tissue types. More than 100 BisAbs have entered clinical trials as of early 2021 for conditions such as cancer and rheumatoid arthritis, utilizing at least 19 different protein architectures binding two or more surface antigens [2]. Recently, bispecific antibodies have garnered favor for activating effector CD4/CD8 T cells through engagement of the CD3 antigen, ubiquitously expressed as a component of the T-cell receptor [3]. Engagement with CD3 through BisAb crosslinking not only co-localizes the T cell to the target cell, but simultaneously activates the effector cell and stimulates proliferation [4, 5]. This engagement facilitates a T-cell mediated immune response that corelates with tumor cell clearance through direct tumor cytotoxicity or activation of apoptotic cell pathways [5].

In many ways, the prototype bispecific T-cell engager antibody is blinatumomab (blina), an αCD3xαCD19 BiTE targeting B cells expressing CD19. Blina is FDA approved for pediatric and adult ALL but has also demonstrated efficacy in other hematological malignancies [6, 7]. Despite a high initial overall response rate, many patients relapse or become refractory to treatment [8]. Reduction in response may be due to low CD19 antigen expression or antigen loss from alternative splicing, non-functional membrane chaperone proteins, transformation to myeloid lineages, or CD19 mutations [9, 10]. Similar mechanisms of resistance develop in response to CD19-targeted CAR-T therapeutics [11]. Because of these changes, CD19-directed therapies in the relapsed/refractory (R/R) setting may demonstrate limited efficacy with eventual relapse in most patients. Further, blinatumomab therapy presents neurotoxicity or cytokine release syndrome risk to some patients [12]. Several more recent alternative BiTEs target B-cell-surface antigens such as CD20, CD38, and B-cell maturation antigen (BCMA). Some of these have entered clinical trials for treatment of B-cell lymphomas, multiple myeloma, and leukemias [2], with some reports of significant efficacy in early trials [13, 14].

Another attractive target in hematological malignancies is CD22, which is broadly expressed on B-cells [15–17]. CD22 is a trans-membrane glycoprotein with internal tyrosine inhibitor motif (ITIM) domains and external sialic acid binding ligand. In B-cells, it functions generally in B-cell receptor (BCR) signaling to inhibit activation through recruitment of SHP-1 and other phosphatases to the cell membrane [18]. We and others have developed various CD22-targeted therapies using constructs such as monoclonal antibodies, antibody drug conjugates (ADCs), and more recently CD22 CAR-Ts [19–24].

CD22-directed treatments have potential to serve as alternatives or complements to CD19- and/or CD20-targeted therapies. Additionally, they can be a treatment option for patients who no longer respond to drugs targeting those antigens. Indeed, in one trial, patients relapsed or refractory to blinatumomab who were then treated with the CD22 ADC inotuzumab experienced 68% complete response as a bridge therapy to hemopoietic stem cell treatment (HSCT) [25]. Additionally, CD22-targeted CAR-T therapies have demonstrated sustained complete responses in patients previously treated with blina or CD19 CAR-T [26].

Here, we describe the initial characterization of a novel αCD3xαCD22 BiTE antibody that has significant in vitro and in vivo activity against ALL and may synergize with blinatumomab. Our best candidate construct performed similarly to blinatumomab in in vitro cytotoxicity studies using ALL/NHL cell lines. Additionally, our CD22 BiTE, at equivalent molar dosages to blinatumomab, demonstrated activation of effector T-cells at levels comparable to blina. Finally, our in vivo data suggests that our construct prolongs survival of mice with ALL xenografts, with minimal toxicity, and demonstrates performance equal to, or exceeding, blinatumomab. We further found that a combination of αCD3xαCD22 and blinatumomab exhibits an enhanced anti-tumor effect in our mouse model, warranting further investigation of dually targeting CD19/CD22 through T-cell engaging treatment modalities. Thus, our novel αCD3xαCD22 BiTE may offer an alternative or companion therapy to CD19-based treatments that can be used in lymphoma therapeutic courses.

## Materials and methods

### Cell lines, antibodies, reagents

The following cell lines were obtained: Raji (ATCC, CCL-86), NALM-6 (DSMZ, ACC 128), K562 (ATCC, CCL-243), U2973 (DSMZ, ACC 642), and Expi293F cells from Expi293 Expression System Kit (Gibco, A14635). RPMI (Gibco, 21-870-076) was supplemented with Fetal Bovine Serum (FBS) (Gibco, A3160402) to 10%, Glutamax (Gibco, 35050061) to 20 mM, sodium pyruvate (Gibco, 11360070) to 1 mM, HEPES buffer (Gibco, 15630080) to 10 mM, 4.5 g/L glucose, and 5 mL of penicillin/streptomycin (Gibco, 15140122) and was used to maintain Raji, NALM-6, and U2973 cells. DMEM (Gibco, 11960044) media was supplemented with 10% FBS and pen/strep and was used for maintaining K562 cells. Expi293F media was used for maintaining Expi293F cells according to manufacturer’s protocol. The following antibodies were obtained for analysis via flow cytometry: anti-CD3-A488 (Clone OKT3, eBioscience), anti-His-PE antibody (clone J095G46 Biolegend), anti-CD22-BV421 antibody (Clone HIB22 Biolegend), anti-CD69-APC Antibody, (Clone FN50 Biolegend), and anti-CD25-BV650 antibody (Clone PC61, Biolegend). Zombie NIR fixable viability kit (Biolegend, 423106) and CFSE (eBioscience, 65-0850-84) were used in live/dead staining for cellular cytotoxicity analysis. Blinatumomab (Blincyto®) was obtained from Amgen (Thousand Oaks, CA). IL-2 (rhIL-2, Biological Resources Branch, NCI, Frederick, MD) was used selectively for in vitro assays.

### Generation of bispecific constructs and purification

Hexahistidine (6xHis) or human influenza hemagglutinin (HA) tagged αCD3xαCD22 BiTE constructs were designed with two single chain variable regions (scFv) for αCD3 and αCD22 with various linker sequences to determine an ideal scaffolding format. The construction of all BiTEs was based upon a similar protocol as described by Loffler et Al. [27]. The αCD3 binding amino acid sequence was derived from the heavy and light chains of the well-characterized CD3 antibody TR66, with a 4xGGGGS linker between the two chains. The αCD22 sequence was derived from a well-characterized αCD22 blocking antibody, HB22.7 [28]. Three proposed constructs with the same binding domain sequences were generated with different orientations of the linker position, composition, and 6xHis or HA tag. Constructs were cloned into pcDNA3.1 + /C-(K)-DYK vector (GenScript, OHu26320D) for expression in Expi293F cells and secretion in cell supernatant. Expi293F cells were transfected using ExpiFectamine (Gibco, A14524) following the manufacturer’s recommended protocol. Antibody presence in supernatant was detected using Coomassie blue stained SDS-PAGE gels and western blots. Supernatant was harvested for purification via Ni–NTA agarose (QIAGEN 30210) or HA agarose affinity columns (Sigma-Aldrich, A2095-1 ml) depending on the respective tag in the construct. Protein concentrations were confirmed using BCA assays (Pierce, 23235) and constructs were buffer exchanged using Amicon spin columns (Millipore, UFC501096) before adding protein stabilizer (2% V/V) used in commercial blinatumomab formulations (Amgen, Thousand Oaks).

### Binding characterization of constructs by flow cytometry

FACS Fortessa 2000 flow cytometer at the UC Davis Medical Center Flow Cytometry Shared Resource (FCSR) core facility was used to characterize binding of αCD3xCD22 constructs to CD22 and CD3 targets. Non-Hodgkin’s Lymphoma (NHL) cell line Raji (CD22-positive control), T cells (CD3-positive control) isolated from donor peripheral blood mononuclear cells (see below for methods) and K562 cells transduced with a CD22 expression cassette were used as controls. Cells were washed with PBS and incubated with each construct for 30 min, then washed again with PBS before 30-min incubation with appropriate secondary antibody. ZNIR was used to identify live cells. Binding was reported in singlet, ZNIR-cell populations.

### Isolation of PBMCs from blood and T-cells from PBMCs

Trima Leukocyte Reduction Chambers from healthy donors were purchased from Vitalant. Peripheral blood mononuclear cells (PBMCs) were isolated from the buffy coat of whole blood using Ficoll-Paque Premium (GE Healthcare Biosciences). PBMCs were incubated in RPMI media prepared as described above at 37° and 5% CO_2_. CD3 + T-cells were isolated from PBMCs using MACS Pan T-cell isolation kit (Miltenyi, 130-096-535) following the manufacturer’s protocol.

### T-cell phenotyping

T cells were isolated from PBMCs that had been thawed and rested overnight in cytokine-free media. The T cells were incubated with CFSE-labeled NALM-6 or U2973 target cells and treated with BiTE 197, blina, or 197 + blina. After 24 h, live T cells (distinguished by ZNIR staining) were analyzed via flow cytometry for activation and proliferation markers CD25 and CD69.

### In Vitro Cytotoxicity assays for ALL/ NHL cell lines

NALM-6 or U2973 cells were CFSE labeled following manufacturer’s protocol and co-cultured as target cells with PBMCs and BiTE constructs to determine enhanced tumor cell killing. PBMCs were rested overnight (16 h) in cytokine-free media prior to initiating killing assays. 50,000 target cells were seeded into 96-well U-bottom plates (Falcon 08-772-3B). PBMCs were added to target cells at various E:T ratios. Single or combined treatments of αCD3xαCD22 BiTE constructs and blinatumomab were added at the indicated concentrations and incubated with PBMC effectors for 24 h. Untreated control groups contained PBMCs but no BiTE treatment. IL-2 was included in some experiments, as indicated, at the designated concentrations. Target cell killing was determined using flow cytometry and analyzed using FlowJo™ v10.8.1 software. Single cell events were determined with side and forward scattering to gate out cell fragments. Live target cells were gated using CFSE live cell staining. Dead cells were gated using Zombie near Infrared (ZNIR) staining. Target cell killing was defined by the percentage of CFSE + single target cells that stained positive for ZNIR.

### In vivo studies: survival and toxicity of NSG mice treated with BiTE constructs

All work with animals was performed in accordance with the national and international guidelines under protocols approved by the University of California Davis Institutional Animal Care and Use Committee (AAALAC accreditation #000029; PHS Animal Assurance #A3433-01; USDA Registration #93-R-0433).

NOD scid gamma (NSG) mice were obtained from Jackson Laboratories and kept at UCDMC vivarium in accordance with Institutional Animal Care and Use Committee (IACUC) protocols until 6 weeks old. All mice were inoculated simultaneously with 10^4^ NALM-6 cells and 10^7^ isolated unstimulated PBMCs combined into a volume of 100uL via subcutaneous (S.C.) injection resulting in an E:T ratio of 1000:1, as previously described [29]. After 1 h, mice were treated intravenously (I.V.) with either blinatumomab (0.1 ug/mouse), BiTE 197 (1 ug/ mouse), or PBS in 100uL aliquots. Mice received 5 total treatments of the same dose on days 0, 1, 2, 3, 4. Blood was collected and pooled from saphenous vein bleeds from two mice per group weekly for 3 weeks for assessment of hematologic, hepatic, and renal toxicity. Blood work analysis was performed by the Comparative Pathology laboratory core at UC Davis Veterinarian Medicine at baseline, day 8 and day 15 after first treatment. Mice were weighed twice weekly and sacrificed upon greater than 15% weight loss from start of treatment or when hind limb paralysis occurred.

This same study was repeated, except in study 2, NALM-6 cells were delivered as xenograft I.V. through the tail vein. In this study, the synergistic effect of BiTE combination was assessed with the addition of a group treated with both blinatumomab (0.1 ug/mouse) and BiTE 197 (1 ug/mouse). Control group (PBS), blinatumomab, and BiTE 197 groups were repeated as described above.

### Statistical analysis

Graphical and statistical analysis were performed using GraphPad Prism 9.1 (GraphPad Software Inc. San Diego, CA). In vitro cytotoxicity assays were analyzed using unpaired students t test with Welch correction. Significance between T cells activated with BiTE 197 or blina was determined using 1-way ANOVA with Tukey’s multiple comparison. In vitro assays were repeated at least twice (2 biological replicates) with 2–3 technical replicates to confirm significance of results. 3 separate PBMC donors were used to evaluate cytotoxicity, and 2 donors were used to evaluate T-cell activation markers. Figures were created from a representative replicate. Log rank mantel-cox significance test was performed to determine whether treatment groups in vivo were significantly different.

## Results

### Construction, validation, and in vitro characterization of an αCD22xαCD3 bispecific antibody

For construction of our αCD22xαCD3 BiTEs, we chose to emulate the overall framework of blinatumomab. Blina’s relatively simple architecture (Supplementary Fig. 1) consists of the two scFVs (for CD3 and CD19), centrally connected by a short flexible linker (GGGGS). The variable heavy and light chains of the two scFv moieties are arranged in the pattern vLvH-linker-vHvL, with flexible glycine-serine linkers between the heavy and light chains. The alternating heavy/light chain pattern is thought to be important to avoid intermolecular mispairing of opposing heavy and light chain fragments [27]. Notably, the absence of any crystallizable fragment (Fc) binding moiety in the BiTE, while allowing for simplicity of construction, is known to severely limit the half-life of the protein in vivo. Therefore, in addition to mimicking blina’s architecture, we explored various central linker modifications that may enhance pharmacokinetics and stability in vivo or provide an additional attachment moiety (handle) to create tri-functional molecules. However, this paper focuses on our best performing candidate, BiTE 197 (which uses a central SGGCGGS linker).

**Fig. 1:**
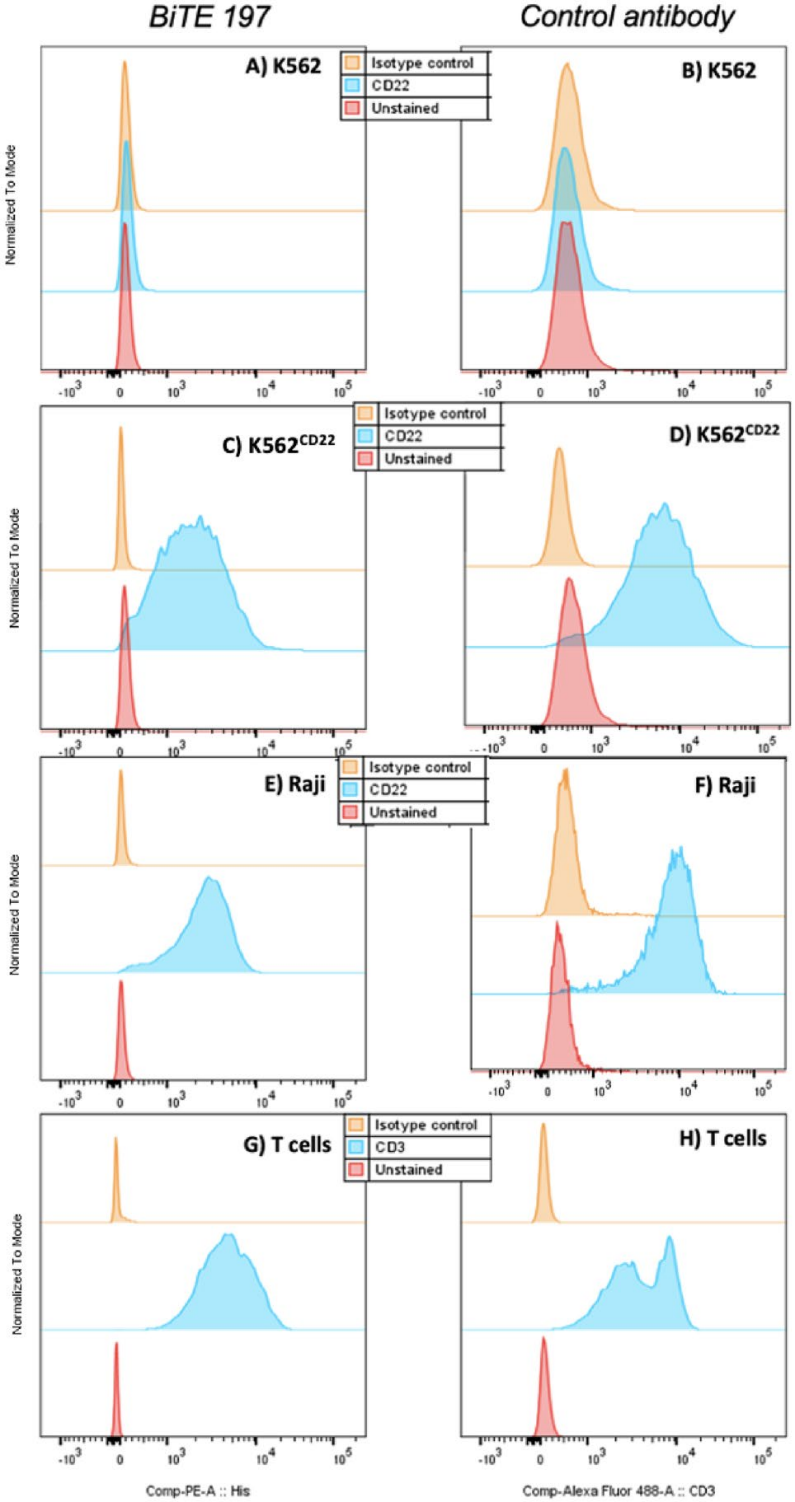
Binding of αCD3xαCD22 BiTE constructs to CD3 or CD22 targets. To test binding of BiTE 197 to its CD22 cognate target, we incubated cells with BiTE 197 at 1 ug/mL and then, probed with a secondary anti-His-PE before flow cytometry analysis. K562 cells do not natively express CD22 as confirmed with a research grade αCD22-BV421 antibody (**B**) and BiTE 197 (**A**). K562 cells were tranduced to express CD22 (**D**), and binding of BiTE 197 was confirmed (**C**). Raji cells natively express surface CD22 in high quantity and BiTE 197 demonstrated binding to Raji cells (**E**) as did a control αCD22-BV421 antibody (**F**). Finally, T cells isolated from healthy PBMCs were incubated with either BiTE 197 then αHis-PE (**G**) or αCD3-AlexaFluor 488 (**H**) to confirm binding to CD3 target

**Fig. 2:**
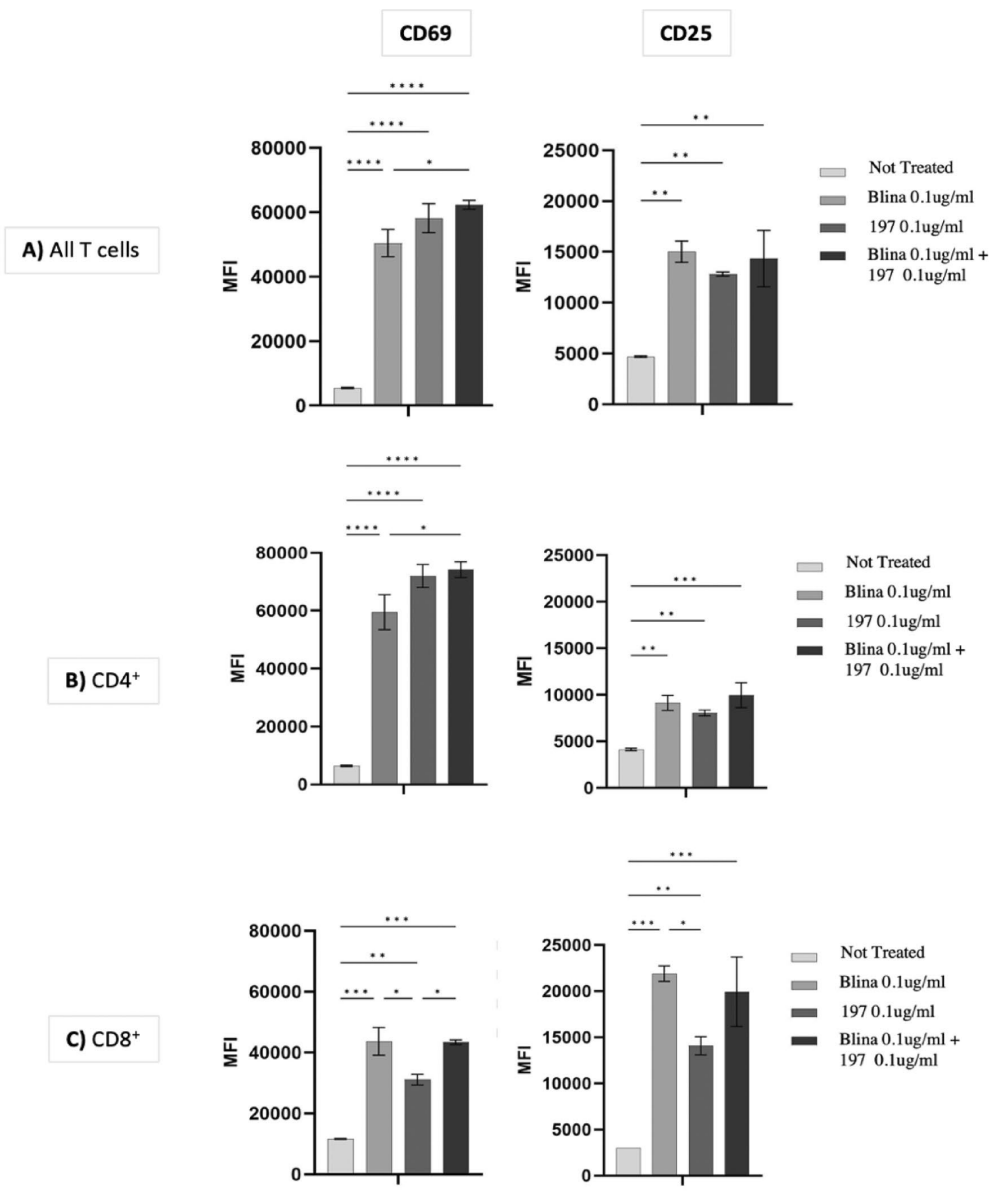
T-cell 24 h activation with CD22 BiTE construct 197. 50,000 T cells were plated with CFSE-labeled target NALM-6 cells at E:T 1:1 and treated with either blinatumomab (0.1 ug/mL), BiTE 197 (0.1ug/mL), Blinatumomab + 197, or target cells alone. Cells were plated without IL-2. Cells were incubated for 24 h then analyzed by Flow Cytometry identifying CFSE- single cells **A** total T cells, **B** CD4, or **C** CD8-gated T cells positive for CD69 and CD25. Median Fluorescence Intensity (MFI) in the T cell population positive for either CD69 or CD25 was used to quantify activation. Significant difference between groups was determined using 1-way ANOVA with Tukey’s multiple comparison test. *p < 0.05; **p < 0.001; ***p < 0.0001; ****p < 0.00001

**Fig. 3:**
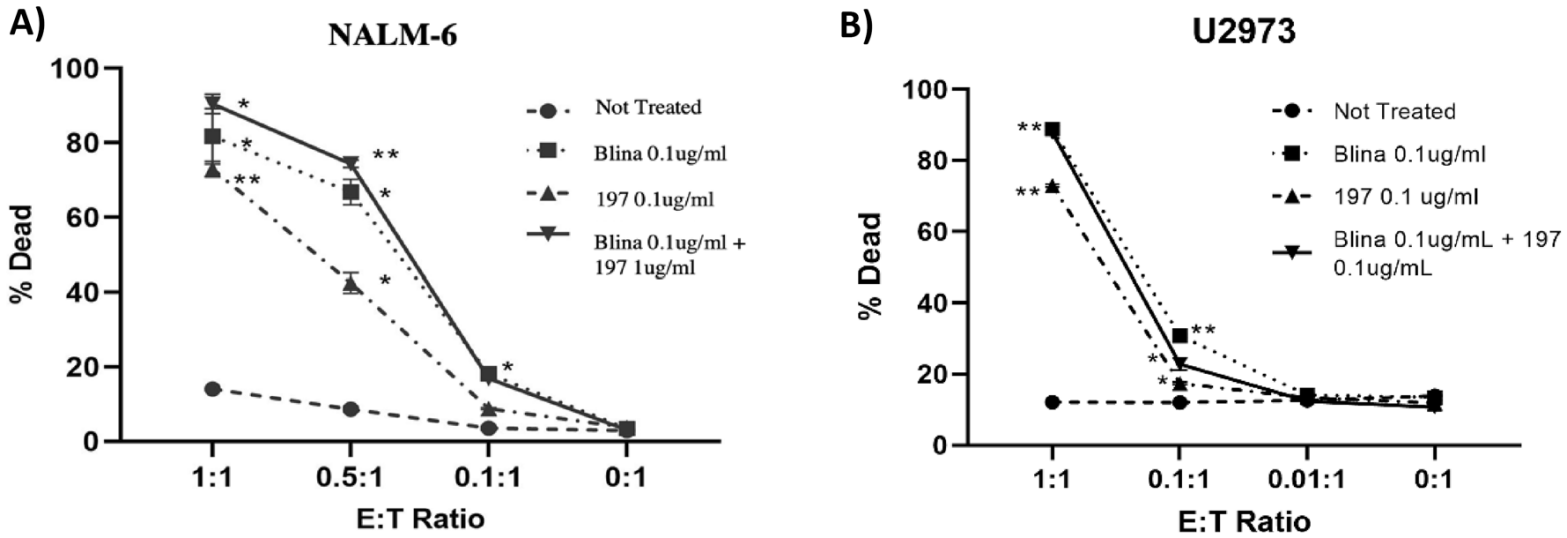
Specific killing of tumor cells in vitro. 50,000 CFSE-labeled target cells were incubated with PBMC effector cells at the indicated E:T ratios and antibody concentrations using **A** NALM-6 and **B** U2973 cells as targets. After 24 h, target cell lysis was measured by flow cytometry. Killing was determined by proportion of cells positive for CSFE/ZNIR out of all CSFE positive cells. All treatment groups were significantly different from the untreated control by unpaired *t* test *p < 0.05; **p < 0.001; ***p < 0.0001

We produced sufficient quantities of our BiTE for all studies by purifying antibody directly from mammalian cell culture (Expi293F) supernatant and purifying with a 6xHis-tag affinity column (Supplementary Fig. 2). We verified binding of each individual arm, in the context of our BiTE, to its cognate target (Fig. 1). While investigation into various linkers is ongoing, we selected the framework for construct 197 (Supplementary Fig. 1) for our in vivo experiments based on in vitro cytotoxicity and T-cell activation at doses and levels comparable to blina (Figs. 2, 3).

### T-cell activation with αCD3xαCD22 BiTE

To assess the effects of BiTE molecules on T-cell activation, we profiled a panel of T-cell phenotypic markers upon exposure to BiTEs 197, blina or 197 + blina in the presence of target cells after 24 h. Our panel included the T-cell activation and proliferation markers CD25 and CD69, which are used extensively to quantify T-cell activation [4, 29, 30]. T-cells were incubated with target tumor cells, with or without BiTE constructs. We found significantly increased BiTE-dependent T-cell activation after 24 h. All treatments increased expression of CD25 and CD69 (Fig. 2). It was notable that T cells treated with BiTE 197 + blina exhibited statistically higher CD69 levels when compared to blina alone, with the CD4^+^ population exhibiting the highest relative increase.

Taken in total, BiTE 197 was comparable to blina in the induction of a rapid and increased T-cell activation as evidenced by increase in CD25 + and CD69 + T cells. This, in addition to the favorable cytotoxicity levels in the dual-treated samples, provided further rationalization for an in vivo assessment of BITE 197.

### Cytotoxicity assays against ALL/NHL cell lines with BiTE molecules

CD22 is expressed broadly on B-cell lymphomas and leukemias and is therefore a suitable alternative target to CD19. Notably, however, CD22 and CD19 display widely different behaviors when it comes to their internalization upon binding (CD22 > > CD19) and the cell-surface density on malignant B-cells (CD19 > > CD22) [32]. These innate differences in the two surface molecules must be considered when comparing of CD19- and CD22-targeted constructs. Nevertheless, we demonstrated that our αCD3xαCD22 BiTE is effective in PBMC-mediated killing of the ALL-cell line NALM-6 and the double hit NHL cell line U2973 at levels comparable to blinatumomab (Fig. 3a, b). Both blina and BiTE 197 demonstrated cytotoxicity greater than 70% against NALM-6 cells at an E:T ratio of 1:1 (Fig. 3a). Additionally, both blina and BiTE 197 killed more than 70% of U2973 cells (Fig. 3b), also at a 1:1 E:T. While several CD22 BiTE constructs were tested (data not shown), BiTE 197 demonstrated superior cytotoxicity in vitro, perhaps due to its short linker length, and therefore, we focused on that reagent for the remainder of this study.

Additionally, we tested the effects of combination treatment with blinatumomab and BiTE 197. At low E:T ratios of 1:1 and 0.5:1, blina killed 82% and 66% of target NALM-6 tumor cells, while BiTE 197 killed 73 and 42% (Fig. 3a). However, the combination of blina and 197 killed 90 and 74% of target cells at the same respective E:T ratios. While not statistically significant, the combination of blina and 197 demonstrated a trend that suggested superiority over blina alone. At the lowest E:T ratio of 0.1:1, the combination treatment killed twice as much as blina (16 vs 8%). These results suggest that the combination is potentially more effective, thus providing further impetus to explore the combinatory effect in vivo*.*

Finally, as part of our cytotoxicity studies, we performed experiments at higher E:Ts, varied antibody concentrations (up to 1 ug/mL for both blina and BiTE 197), as well as with added exogenous IL-2. As shown in Supplemental Fig. 3, the results suggested that in the absence of exogenous IL-2 (as would be the case in vivo), blina efficacy reached an in vitro plateau at 0.1 ug/mL, while BiTE 197 displayed maximal in vitro activity (essentially matching blina) at 1 ug/mL.

### In vivo studies

#### Survival

The ALL-cell line NALM-6 was selected for in vivo murine xenograft studies based on our in vitro cytotoxicity studies as well as previously published work with blina [29]. We chose a blina dose of 0.1ug/mouse, following the protocol established in [29], a study which also demonstrated comparable efficacy of 1 ug and 0.1 ug/mouse dosages. BiTE 197 was administered at a higher dose of 1 ug/mouse because of (a) the known greater propensity of CD22 to internalize upon antibody binding [32] (up to tenfold greater than CD19); (b) the established 5-to-sixfold lower surface expression of CD22 compared to CD19 on NALM-6 cells [31]; and (c) the assumed greater purity and stability of a commercial product (blina) versus our lab-synthesized construct. Based on these combined differences, we reasoned that a BiTE 197 dosage of 1ug/mouse would be appropriate for our in vivo studies.

**Fig. 4:**
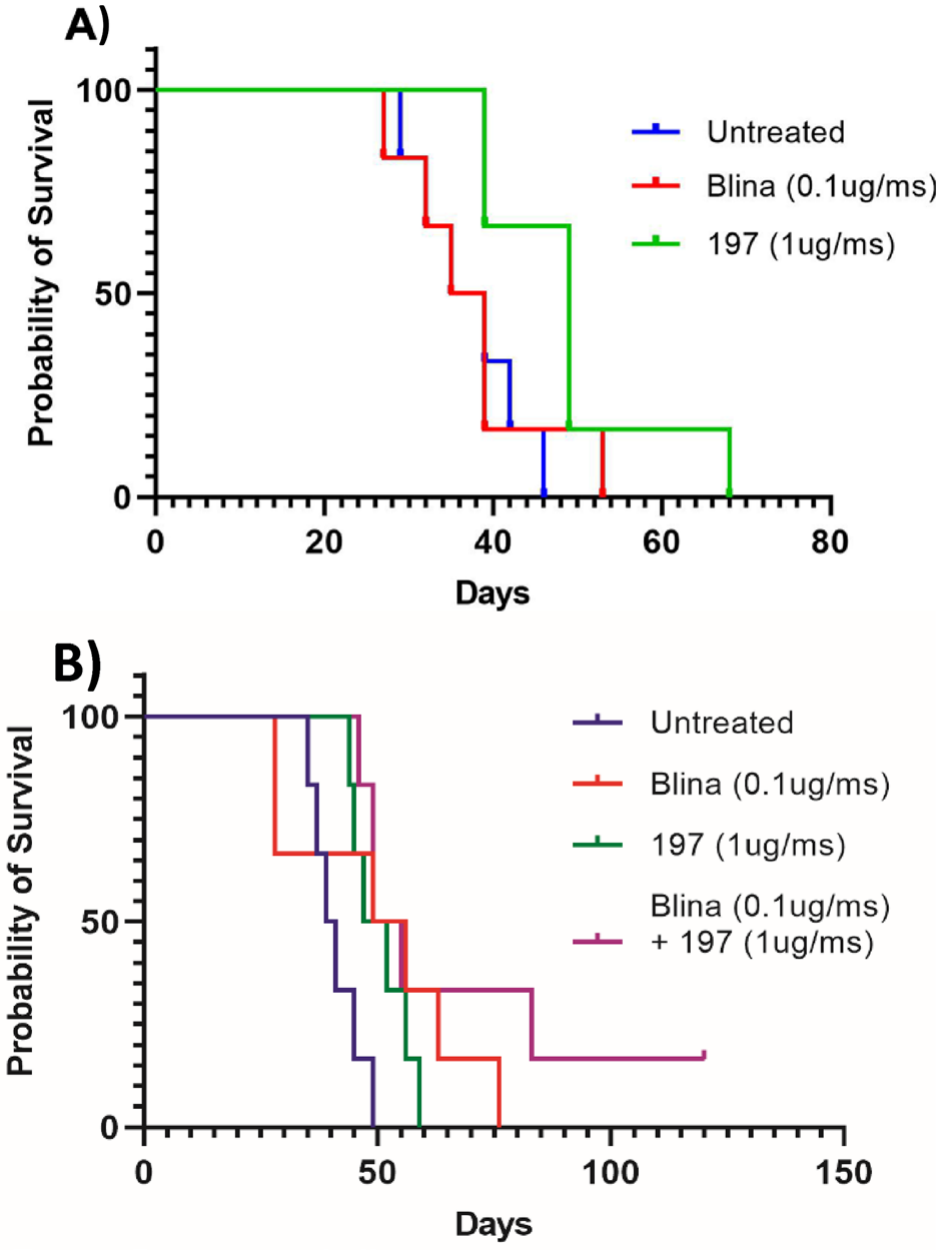
Survival of NALM-6 Xenograft NSG mice. **A** Study 1**:** NSG mice were inoculated with 10^4^ NALM-6 cells and 10^7^ PBMCs through S.C. injection and treated with BiTE construct 197, blinatumomab, or PBS (negative control) daily for 5 days. *N* = 6 for each group. Mice were euthanized upon significant weight loss or hind limb paralysis. **B** Study 2: NSG mice were inoculated with 10^4^ NALM-6 cells and 10^7^ PBMCs IV through tail vein injection and treated with BiTE construct 197, blinatumomab, 197 + blinatumomab, or PBS (negative control) daily for 5 days. *N* = 6 for each group. Mice were euthanized upon significant weight loss or hind limb paralysis. Survival was significantly different between treated and untreated groups by log rank Mantel-Cox significance test

Survival data from studies 1 and 2 are reported in Table 1. In study 1, a subcutaneous leukemia model was used, and 4/6 mice treated with BiTE 197 survived longer than 50 days, while only 2/6 mice treated with blina reached the same milestone (Fig. 4a). To validate the results of study 1 and assess the efficacy of combination therapy, a similar model was established with I.V. injection of tumor cells. In study 2, all treatment groups—blina, BiTE 197, and combination blina + 197—performed similarly with 3/6 mice surviving past day 50 (Fig. 4b) in each group. However, 2/6 mice in the combination treatment group survived longer than the maximal survival time for either blina or BiTE 197 alone (> 76 days), suggesting a possible treatment benefit from concurrent dosing. Further studies are planned to elucidate the mechanism for this potential synergy and confirm its efficacy. All untreated control mice survived less than 50 days in both studies.

**Table 1:** Survival statistics from in vivo (A) study 1 (*p* = 0.0245); (B) study 2 (*p* = 0.0151)

Group	Population size	Median Survival
*(A)*		
Control (pbs)	*N* = 6	37 days (29–46)
Blinatumomab (0.1ug/mouse)	*N* = 6	37 days (27–53)
BiTE 197 (1ug/mouse)	*N* = 6	49 days (39–68)
*(B)*		
Control (pbs)	*N* = 6	40 days (35–49)
Blinatumomab (0.1ug/mouse)	*N* = 6	52.5 days (28–76)
BiTE 197 (1ug/mouse)	*N* = 6	49.5 days (44–59)
Blinatumomab (0.1ug/mouse) + 197 (1ug/mouse)	*N* = 6	52 days (46–120)

Long rank (Mantel Cox) test was used for group difference analysis

### Treatment-related toxicity

Treatment toxicity was assessed in study 1. Hepatic and hematologic toxicity data are presented in Supplemental Tables 1 and 2. No significant treatment-related toxicities were observed with treatment using either 197 or blina. Elevated AST and ALT were observed in the blina treated mice during the 3^rd^ week.

## Discussion

In this study, we demonstrate successful production and characterization of a novel αCD22xαCD3 BiTE using an architecture analogous to the therapeutic agent blinatumomab. Multiple constructs, containing the same binding domains with varying linkers, were developed, but only BiTE 197 is presented here based on its superior in vitro efficacy that compares favorably to blina.

Given its structural similarity to blina, our BiTE, when used as a single agent, would be expected to display many of the same pharmacokinetics as the FDA-approved drug. These include a short in vivo half-life that would likely require continuous IV infusion and a similar dosing schedule. While second- and third-generation BisAbs have demonstrated prolonged retention and more convenient dosing, we chose to retain this format to facilitate comparison with blina. As we have noted, a direct equimolar comparison of dosing between blina and 197 is challenging due to a number of factors, including differential internalization behavior and antigen density of CD19 and CD22 on target tumor cells. In regards to therapeutic usage, as CD19- and CD22-based CAR-T therapies have shown similar side effects in terms of cytokine release syndrome and neurotoxicity [34], it is possible that single-agent therapy with BiTE 197 could present the same spectrum of manageable toxicities as CD19 BiTE.

In clinical studies, blinatumomab typically produces a high initial response rate [6, 8, 33]. However, most patients relapse within 1–3 years depending on factors such as disease severity or number of prior treatments at treatment initiation [9–11, 25]. Additionally, escape strategies in response to CD19-targeted drugs are widely recognized in the clinic [9–11]. Therefore, alternative therapies, either as salvage courses, or potentially in combined regimens, are needed.

Numerous CD19- and CD20-targeted BiTEs have demonstrated killing efficacy across NHL/leukemia subtypes and cell lines [4, 29, 30, 35, 37, 38]. We are aware, however of just one other αCD3xαCD22 BiTE, which uses a humanized heavy chain-only format. This molecule is currently in clinical trials in the USA, but no data have been published [39].

There is both pre-clinical and clinical evidence in support of CD22-directed therapies in NHL and ALL settings [19–22]. Indeed, CD22 is an attractive target due to its ability to internalize when bound by antibody, making it popular target for antibody drug conjugates [31]. Also, the potential benefits for combining CD19 and CD22 (as well as CD20) in antibody and CAR-T format has been demonstrated in lymphoma models [24, 36–38, 40]. Still, we are not aware of concomitant use of BiTEs targeting CD22 and CD19, a novel strategy that we employed here. Interestingly, our in vivo data suggests a possible benefit in mice receiving a combination treatment of BiTE 197 and blinatumomab, supporting the potential usefulness of our construct in a muti-targeted approach. Furthermore, the anti-CD22 scFv used in BiTE 197 has CD22 ligand blocking properties, which may prove efficacious based on previous studies that have shown superior tumoricidal effects when compared to non-ligand blocking anti-CD22 mAbs [21].

In summation, we have presented data that demonstrate merit in utilizing CD22 as an alternate target antigen to CD19 or CD20 in a BiTE formulation for B-cell malignancies. We suggest that our novel construct could be used as a primary, combination, or post-CD19 directed therapy.

### Study limitations

In the production of our BiTEs, there may have been additional non-functional protein fragments or aggregates, evidenced by faint streaks in the coomassie blue stained SDS-PAGE gels (Supplementary Fig. 2). Additionally for this study, we concentrated purified protein from supernatant of mammalian cell culture without additional size-exclusion chromatography to remove non-functional protein fragments, thus likely producing a lower effective concentration for out BiTEs. No pharmacokinetic (PK) analysis was obtained in this study to quantify serum half-life. Previous studies with blinatumomab demonstrate serum clearance half-life in humans of 2.1 h [7]. Future studies with our BiTE could investigate T-cell proliferation in vivo and perform PK studies to determine serum half-life. In our study, no maximum tolerable dose was reached, although no mice were euthanized from study from treatment-related side effects. Finally, as discussed, the BiTE 197 dosage in our in vivo studies was tenfold higher than blina, but confounding differences in CD19 and CD22 expression and internalization suggest that a molar dosage equivalence may not be appropriate and make it difficult to perform direct comparisons regarding treatment efficacy of blina and BiTE 197.

## Supplementary Information

Below is the link to the electronic supplementary material.

Supplementary file1 (DOCX 4226 KB)
