# Supplementary material for: A Novel bispecific T-cell engager (BiTE) targeting CD22 and CD3 has both in vitro and in vivo activity and synergizes with blinatumomab in an acute lymphoblastic leukemia (ALL) tumor model
Source: Cancer Immunol Immunother. 2023 May 29;72(9):2939–48. doi: 10.1007/s00262-023-03444-0 (PMC10412491; doi:10.1007/s00262-023-03444-0)
Supplement: Supplementary file 1 — Supplementary file1 (DOCX 4226 KB) [file 262_2023_3444_MOESM1_ESM.docx]

Cancer Immunology, Immunotherapy – Joshua F Meckler et. al

**A Novel bispecific T-cell engager (BiTE) targeting CD22 and CD3 has both in vitro and in vivo activity and synergizes with blinatumomab in an acute lymphoblastic leukemia (ALL) tumor model**

# Supplemental Figures


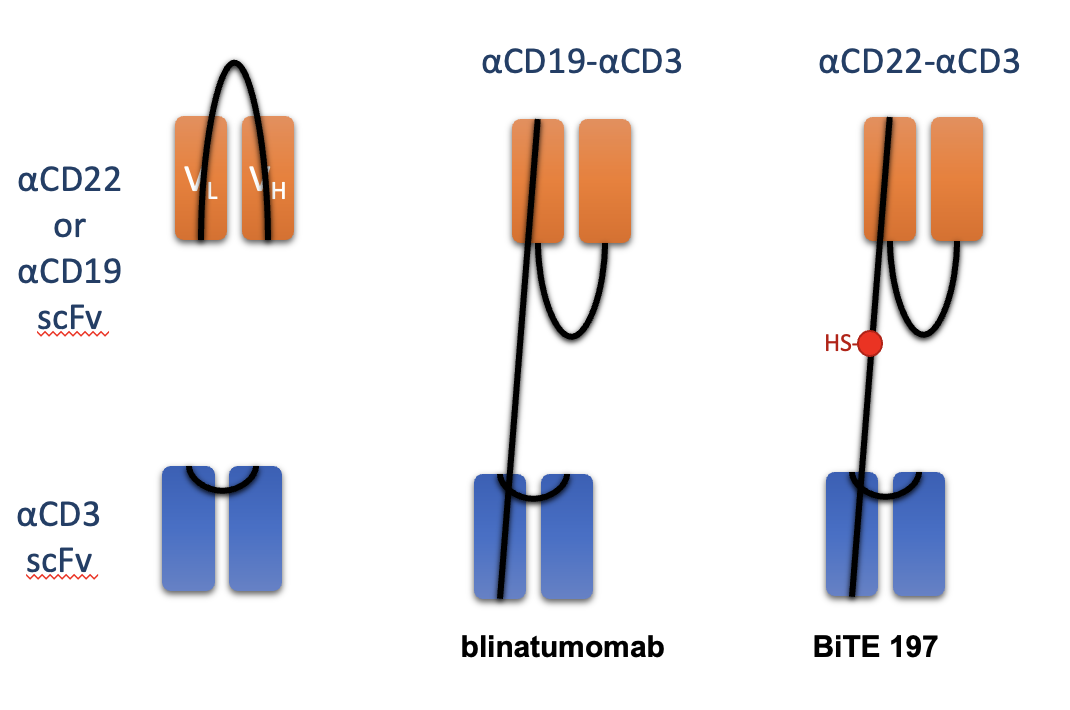


**Supplemental Figure 1. Graphical representation of BiTE constructs.** ScFv fragments for human αCD22 antigen and αCD3 antigen were placed in sequence to produce a BiTE molecule with similar architecture to blinatumomab.

BiTE 197

**
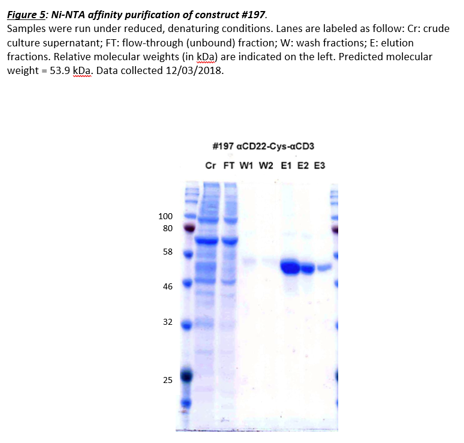
Supplemental Figure 2. Ni-NTA affinity purification of αCD3xαCD22 BiTE construct 197.** Samples were run under reduced, denaturing conditions. Lanes are labeled as follow: Cr = crude culture supernatant; FT= Flow through (unbound) fraction; W= wash fractions; E= elution fractions. Relative molecular weights (in kDa) are indicated on the left. Predicted molecular weight= 53.9 kDa.


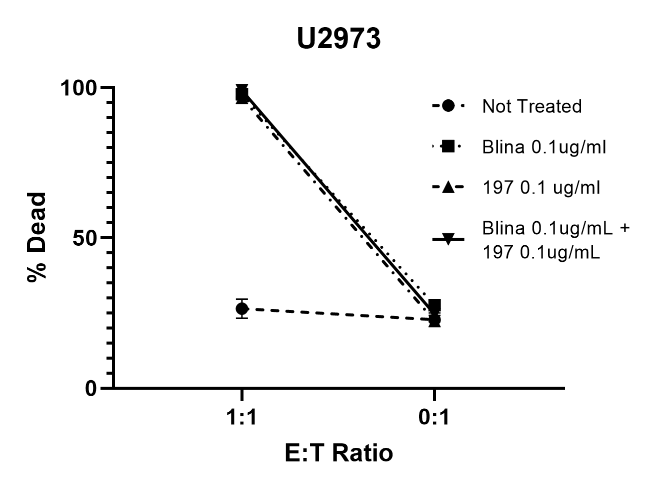

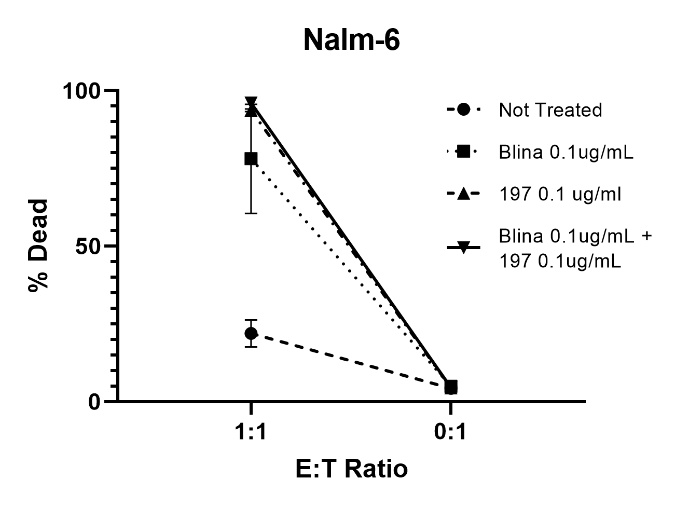

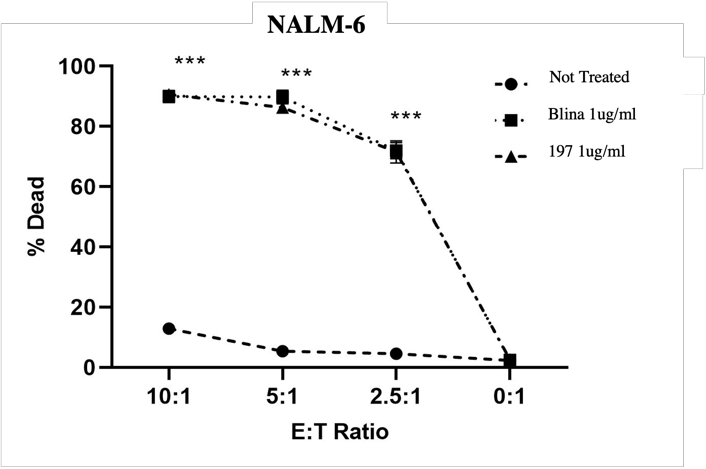

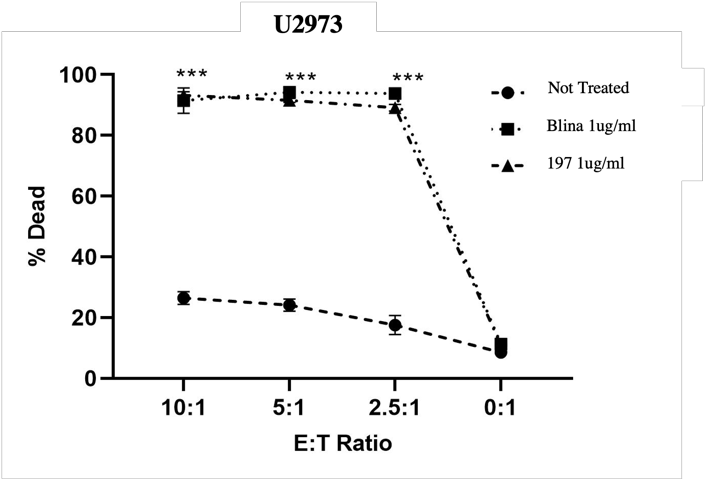


**D)**

**C)**

**B)**

**A)**

**Supplemental Fig 3.** Specific killing of tumor cells in vitro **(A).** 50,000 NALM-6 Cells were incubated with PBMC effector cells at the indicated E:T ratios and antibody concentrations in the presence of 500 U/ mL of IL-2. **B)** the assay was repeated with U2973 cells. (**C and D)** Additional trial with separate PBMC donors using the same conditions as Fig 3a-b in the main body of the paper. Briefly, 50,000 **C)** NALM-6 or **D)** U2973 target cells. No IL-2 was added for the experiments in C and D.


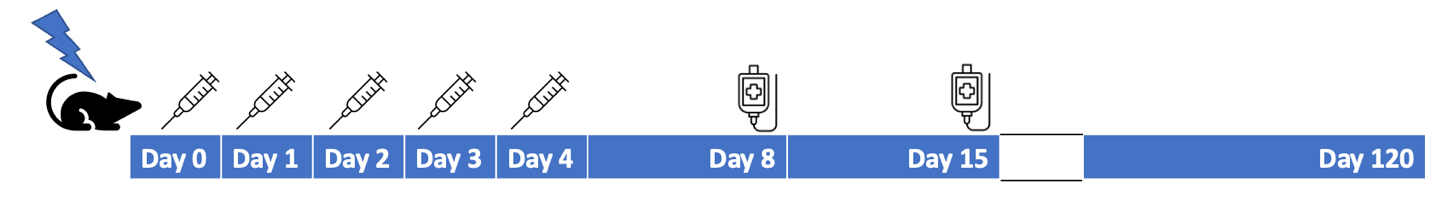


| **Day 0** | 1. Blood drawn from mice for baseline liver/ CBC analysis 2. NSG were inoculated with NALM-6 tumor cells and human PBMCs, sorted into groups and dosed with blinatumomab (0.1ug/mouse), BiTE 197 (1ug/ ms), Blina + BiTE 197 (total 1.1 ug/ ms), or pbs delivered IV. |
| --- | --- |
| **Day 1-4** | Mice receive treatment |
| **Day 8, 15** | Blood drawn from tail vein from 1 mouse/ group for treatment toxicity analysis |
| **Day 120** | Study complete, remaining mice euthanized |

**Supplemental Figure 4.** **Dosing schematic for *in vivo* mouse studies**. NSG mice were sorted into groups of 6 for αCD3xCD22 BiTE construct, blinatumomab, or pbs (control). Mice were radiated before tumor implantation on day

0.

**
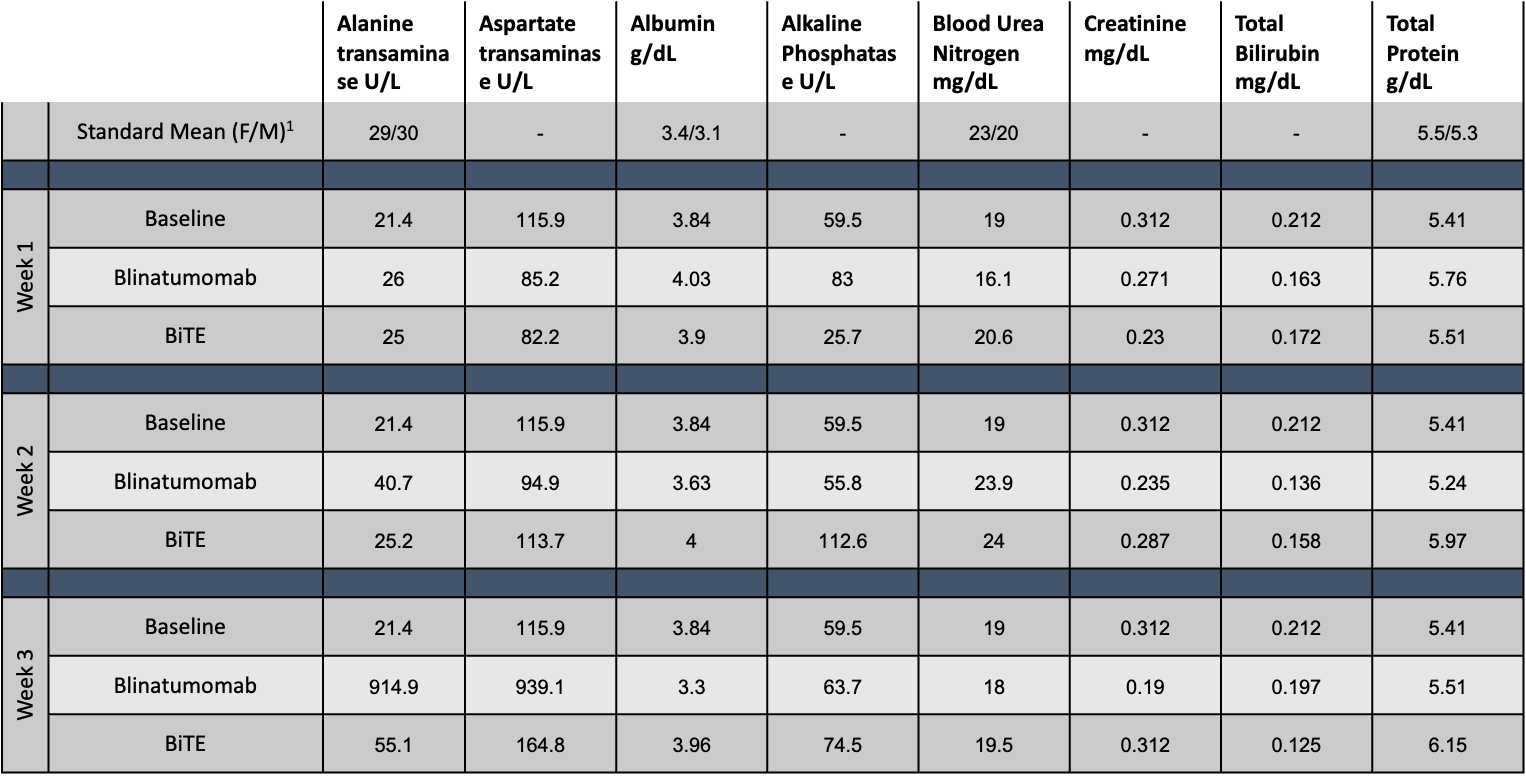
**

1. Jackson lab summary of physiology data for NOD.Cg-Prkdc^scid^ Il2rg^tm1Wjl^/SzJ at 8 weeks

**Supplemental table 1a. Blood panel to detect treatment mediated toxicities**. Baseline value was taken before xenograft transplantation and is listed in each time period for reference only. Mice treated with either blinatumomab or BiTE were examined weekly post treatment for the first 3 weeks. Each reading represents sample from one mouse selected at random at each time interval within appropriate treatment group.

|  | Standard Mean (Female/Male)^1^ | Baseline | Blinatumomab | | | | | BiTE construct | | | | |
| --- | --- | --- | --- | --- | --- | --- | --- | --- | --- | --- | --- | --- |
|  |  |  | W1 | W2 | | | W3 | W1 | | W2 | W3 | |
| WBC (K/ul) | 0.64/0.96 | 2.56 | 2.80 | 3.34 | | | 6.18 | 1.44 | | 2.38 | 2.76 | |
| Absolute Neutrophil cells (K/ul) | 0.44/0.59 | 2.02 | 1.99 | 2.97 | | | 5.63 | 1.13 | | 2.08 | 2.53 | |
| Absolute Lymphocyte cells (K/ul) | 0.11/0.59 | 0.40 | 0.47 | 0.22 | | | 0.21 | 0.23 | | 0.19 | 0.14 | |
| Absolute Monocyte cells (K/ul) | 5.8/10.7 | 0.06 | 0.13 | 0.13 | | | 0.16 | 0.04 | | 0.09 | 0.04 | |
| Absolute Eosinophil cells (K/ul) | 0.05/0.07 | 0.07 | 0.12 | 0.01 | | | 0.16 | 0.03 | | 0.01 | 0.04 | |
| Absolute Basophil cells (K/ul) | 0.01/0.00 | 0.01 | 0.08 | 0.01 | | | 0.03 | 0.01 | | 0.00 | 0.01 | |
| Neutrophil % | 67.5/61.9 | 78.96 | 71.23 | 88.89 | | | 91.07 | 78.65 | | 87.33 | 91.74 | |
| Lymphocyte % | 16.8/19.7 | 15.67 | 16.84 | 6.69 | | | 3.33 | 15.79 | | 7.96 | 5.04 | |
| Monocyte % | 5.8/10.7 | 2.30 | 4.53 | 3.89 | | | 2.61 | 2.96 | | 3.94 | 1.59 | |
| Eosinophil % | 9.1/7.3 | 2.62 | 4.46 | 0.37 | | | 2.56 | 2.00 | | 0.56 | 1.28 | |
| Basophil % | 1.1/0.6 | 0.45 | 2.94 | 0.16 | | | 0.43 | 0.60 | | 0.20 | 0.36 | |
| RBC (M/ul) | 8.78/9.08 | 10.12 | 9.18 | 8.53 | | | 9.28 | 9.81 | | 8.75 | 9.17 | |
| Hemoglobin (g/dL) | 14.0/14.3 | 14.6 | 13.6 | 14.0 | | | 13.7 | 14.9 | | 13.3 | 13.6 | |
| Hematocrit % | 44.2/45.3 | 49.6 | 46.4 | 45.2 | | | 45.3 | 50.5 | | 42.7 | 45.8 | |
| MCV (fL) | 50.4/49.9 | 49.0 | 50.5 | 53.0 | | | 48.8 | 51.5 | | 48.8 | 50.0 | |
| MCH (pg) | 16.0/15.8 | 14.4 | 14.8 | 16.4 | | | 14.8 | 15.2 | | 15.2 | 14.8 | |
| MCHC (g/dL) | 31.8/31.6 | 29.4 | 29.3 | 31.0 | | | 30.2 | 29.5 | | 31.1 | 29.7 | |
| RDW % | - | 19.9 | 20.1 | 21.4 | | | 20.1 | 20.5 | | 19.6 | 20.0 | |
| Platelets (K/uL) | 1192/1340 | 1116.0 | 1000.0 | 1476 | | | 453 | 717.0 | | 1356 | 904 | |
| MPV (fL) | 4.6/4.6 | 5.6 | 5.7 | | 5.3 | 6.2 | | 5.8 | 5.3 | | | 5.7 |

Jackson lab summary of physiology data for NOD.Cg-Prkdc^scid^ Il2rg^tm1Wjl^/SzJ at 8 weeks

**Supplemental table 1b. Full CBC for treated NALM-6 xenograft mice.**
